# Legitimizing neglect - a qualitative study among nursing home staff in Norway

**DOI:** 10.1186/s12913-023-09185-1

**Published:** 2023-03-06

**Authors:** Stine Borgen Lund, John-Arne Skolbekken, Laura Mosqueda, Wenche K. Malmedal

**Affiliations:** 1 Department of Public Health and Nursing, Norwegian University of Science and Technology (NTNU), PO Box 8905, Trondheim, 7491 Norway; 2 Keck School of Medicine, University of Southern California, Los Angeles, USA

**Keywords:** Neglect, Nursing home, Residential care, Long term care, Elder abuse, Elder neglect, Missed care, Rationing of care, Nursing staff`s perceptions, Qualitative, Constructivist grounded theory

## Abstract

**Introduction:**

Residents in nursing homes do not always get qualitatively good nursing care, and research shows that residents’ basic care needs are sometimes neglected. Neglect in nursing homes is a challenging and complex issue, yet a preventable one. Nursing home staff are at the frontline of detecting and preventing neglect but may also be the ones causing it. It is essential to understand why and how neglect happens in order to recognize, expose, and prevent its occurrence. Our aim was to generate new knowledge on the processes leading to and allowing neglect to continue in Norwegian nursing homes, by studying how nursing home staff perceive and reflect on when nursing home residents are neglected in their daily practice.

**Methods:**

A qualitative exploratory design was used. The study was based on five focus group discussions (20 participants, total) and ten individual interviews with nursing home staff from 17 different nursing homes in Norway. The interviews were analysed according to Charmaz constructivist grounded theory.

**Results:**

In order to make neglect an acceptable practice, nursing home staff apply different strategies. These strategies were identified as when the staff legitimize neglect by *neglecting neglect*, when the staff are not recognizing their own behaviour as neglectful, as expressed in their actions and language, and *normalizing missed care* when resources are lacking and nursing staff are rationing care.

**Conclusions:**

The gradual shift between judging actions as neglectful or not are made possible when nursing home staff legitimize neglect by not recognizing their practice as neglective, thus neglecting neglect or when they are normalizing missed care. Increased awareness and reflections on these processes may be a way of reducing the risk of and preventing neglect in nursing homes.

**Supplementary Information:**

The online version contains supplementary material available at 10.1186/s12913-023-09185-1.

## Introduction

Professional nursing care is often presented through idealised descriptions where carers provide holistic patient centred care covering the patients’ physical, psychological, social, and spiritual needs [1]. When it comes to actual practice, here is often a discrepancy between these ideals and the reality of practice, particularly in the nursing home setting. This paper explores one particular discrepancy, that of unmet needs which are referred to as neglect. Examples include: putting a resident in a diaper rather than helping them to the bathroom on a regular basis, ignoring residents who are viewed as demanding or agitated, and lack of attention to a residents’ need for physical activity and social stimuli [2–9].

These practices are clearly contrary to the expectations of good nursing practice and are contrary to the best interest of the residents [10], relatives [11] and staff [12–14]. They represent a significant and preventable problem that stems from a variety of causes including personal attributes, interpersonal relationships, systems issues, and moral/ethical/cultural considerations.

In line with the constructivist grounded theory approach guiding our work [15], we sought to understand the end result of neglect in nursing homes as a result of social processes and interactions among the staff members, administrators, families, and residents that then shape an individual staff member’s views and behaviours. In doing so, we acknowledge the reality of the suffering of nursing home residents, the challenges faced by the nursing home staff, that this reality is co-constructed by many contributors, and that it has real consequences.

We thus approach this field of research from a moderate constructionist position [16]. By doing so, we do not take neither neglect nor human needs for granted, to be studied as natural kinds. In this paper, we use the same concepts as other researchers in the field, based on a constructionist epistemology. We treat them as part of a negotiable reality, that serve us as humans in our meaning making of the world. This meaning making has been framed in different discourses by various researchers, as presented in the background section below. In this paper, we analyse the meaning making of nursing home staff, as a co-construction of nursing home reality, in an effort to contribute to the research-based dialogue that eventually can aid the reduction of human suffering.

Our aim in this paper was to generate new knowledge on the processes leading to and allowing neglect to continue in Norwegian nursing homes, based on the research question “How do nursing homes’ staff perceive and reflect on neglect?” We argue that understanding how nursing home staff conceptualize neglect is a crucial step in moving towards solutions. By doing so, we have generated knowledge on how neglective nursing practices exist and prevail in Norwegian nursing homes.

### Background

A challenge throughout this study has been to define and reach agreement on the concept of unmet needs which we are choosing to call neglect. Different concepts and different definitions have been used to explain the what, how, and why when an older person is harmed or put at risk for harm, by an active or passive act, intentional or unintentional, by a person or a system. Below we present some terms frequently used to label these situations.

Elder abuse has been discussed, defined, and conceptualized by many different disciplines including gerontology, criminology, social work, social welfare, law, sociology, psychology, and medicine [17]. Different disciplines add new perspectives and new lenses through which we can view elder abuse: as a public health issue, a medical syndrome, a crime, and a human rights violation are some examples. In fact, these are not mutually exclusive or competing views rather they may all be true simultaneously and to different degrees under different circumstance.

In 2016 the Centers for Disease Control and Prevention (CDC) defined elder abuse as “*An intentional act or failure to act by a caregiver or another person in a relationship involving an expectation of trust that causes or creates a risk of harm to an older adult.*” A subtype of elder abuse is neglect, defined by the CDC as “*Failure by a caregiver or other person in a trust relationship to protect an elder from harm or the failure to meet needs for essential medical care, nutrition, hydration, hygiene, clothing, basic activities of daily living or shelter, which results in a serious risk of compromised health and/or safety, relative to age, health status, and cultural norms*” [18].

Rarely discussed in the context of elder abuse are the terms *missed care* and *missed nursing care* yet both are relevant and relate directly to neglect [19]. *Missed care* is derived from the idea of *task left undone*, first introduced by Aiken and colleagues in 2001 [20]. Kalisch claim that the first identification of missed nursing care was in her qualitative study from a hospital setting in 2006. Nursing staff categorized the reasons for missed nursing care to staff shortages, poor use of existing staff resources, time required for the nursing intervention, poor teamwork, ineffective delegation, habit, and denial [21]. Other terms such as unfinished care and prioritization are sometimes used to describe the same phenomenon that we refer to as neglect [9]. In biomedical ethics, the terminology *health-care rationing* is used, and in medical and health care quality literature, the term *underuse* is common [22]. The concept *error* is depicted in the patient safety literature and defined as “*an act of commission (doing something wrong) or omission (failing to do the right thing) leading to an undesirable outcome or significant potential for such outcome*” (ibid. p:19). This too relates to missed care and neglect.

A broadening of the CDC definition of neglect to include any aspect of required patient care that is omitted (either in part or in whole) or delayed may thus become viable. This includes the provision of basic care for physical needs, psychological support, and attending to the emotional needs of each patient [5, 23–25]. It further refers to acts of omission, which have received less attention than acts of commission within the patient safety literature [26, 27].

Despite the different types of neglect presented, there is agreement on most of the signs, consequences, and outcomes of neglect; these are both physical and emotional/psychological [28]. Physical outcomes of neglect can be bedsores, malnutrition, dehydration, poor hygiene/infections, contractures, injuries related to falls, incidents or adverse events resulting in patient harm and in worst-case premature death [6, 28–31]. Emotional and psychological outcomes can be related to change in self-perception, expressed as depression, fear, anxiety, anger, irritability, even suicidal thoughts, and compromised quality of care [6, 28, 32]. Neglect of nursing home residents may have devastating consequences due to the multimorbidity, frailty and cognitive impairment of this patient group. Hence, minor incidents or neglected care needs have major consequences when repeated over time [12, 24], contributing to neglect as a patient safety issue [7, 9, 33, 34]. Signs of neglect in nursing homes residents can often be hidden by the natural aging process, hence difficult to recognize and acknowledge [31].

When explaining why *abuse and neglect* and *missed care* occur, the literature around elder abuse in nursing homes originally focused on three key factors: environmental conditions, staff characteristics, and resident characteristics [14, 35]. More recently, more comprehensive models have been introduced, like ecological models where individual risk factors (characteristics related to both the patient and the care professional), relational risk factors (relationship between staff and resident), institutional risk factors (e.g. high workload/stress, management, staffing, safety culture), and societal factors like cultural norms, public awareness, and ageism are included [36–39]. An overview of reviews on missed nursing care in hospital settings concluded that missed care is related to staffing levels and/or labour resources, skill- mix, lack of material resources, patient acuity and teamwork/communication [40]. It is not clear to what extent findings in hospital settings apply to nursing home settings. While these two settings have some commonalities there are also differences in how they are structured, funded, staffed, and operated. Hence findings from acute care may not be generalizable to nursing homes [9]. Campagna, et al. (2022) on the other hand, concluded that missed care in hospital environments are relevant for nursing homes due to similarities such as patients with severe and complex clinical conditions, high perceived workload, and less qualified staff [41].

In this study we explore the nursing home environment, studying how nursing home staff perceive neglect and how they react when given an opportunity to reflect on scenarios that describe nursing home residents with unmet needs. We do this in a Norwegian context, which is briefly described for international readers below.

### The Norwegian nursing home context

In Norway, there are approximately 40 000 (13% of the population > 80 years) nursing home residents, with a mean age of 85 years. Most of them have complex medical conditions requiring close monitoring/follow-up and help in activities of daily living. Over 80% have dementia with accompanying neuropsychiatric symptoms such as agitation, aggression, depression and anxiety [42].

The majority of Norwegian nursing homes are run by municipalities and financed by service fees and taxes. A small percentage of Norwegian nursing homes are managed by private for-profit agencies (5%) or non-profit organizations (5%) [43]. The Ministry of Health and Care Services provides specific regulations regarding leadership, staffing and residents´ rights in Norwegian nursing homes. The provision of care is delivered under the National Regulation of Quality of Care in an attempt to ensure that residents’ basic care needs including psychological, physical, psychological, and social needs are met [44]. There are no mandatary staff-to resident ratio or standards for staff´s qualifications [45]. Nor are there any requirements with regard to skill-mix.

The nursing home staff who provide direct patient care include registered nurses (RN), licensed practical nurses (LPN), assistants with no formal education, and social educators/social workers (SE/SW) [46]. Due to a shortage of nurses (RN), recruitment problems, and challenges of keeping nurses (RN) in nursing homes, a high number of unskilled personnel are hired. Assessment of adequate staffing and skill-mix are in the hands of individual nursing home managers or nurses in charge. Norwegian nursing homes are characterised by lack of competent personnel, high workload, and high sick-leave of carers, all of which have a negative effect on quality of care [47].

## Method

This study is based on the guidance given for a constructivist grounded theory (CGT) study [15]. Accordingly, data collection and analysis were performed in an iterative process. Data were gathered through a combination of focus groups discussions (FG) and individual interviews (II). Focus groups were chosen initially based on its potential for producing rich data, but also for logistic reasons. After initial analyses they were supplemented with individual interviews, primarily to check the possible negative effect of group interactions when addressing a potentially sensitive issue. Both focus groups discussions and individual interviews were based on semi-structured interview guides, which were developed and adjusted in line with our analyses (Additional file 1). Interview guides were also supplemented by case descriptions and statements about elder neglect from a survey instrument on elder abuse in Norwegian nursing homes [4] during the data collection. These inputs were introduced late in the focus groups/interviews, after the participants’ spontaneous responses had been explored. Focus group discussions lasted approximately 90 min, while individual interviews lasted from 55 to 90 min. All interviews were audiotaped and transcribed verbatim by an experienced transcriber (HF).

### Participants

Recruitment for this study is based on sampling through nursing homes, educational settings and individual gatekeepers. Participants were recruited over a 17- month period from April 2019 to November 2020. The Covid pandemic interfered with our recruitment from March 2020, as nursing home staff were not easily available in this period. A total of 30 nursing home staff from 17 different nursing homes, four rural and 13 urban from municipalities in middle Norway participated in five focus group discussions and ten individual interviews. Persons who have or have had experience in providing basic care to residents in long-term nursing homes were included in the study, across the variety of professions qualifying for such jobs. An overview of the interview data is presented in Table 1.

**Table 1:** Overview of interview data

Interview	RN	LPN	Ass	Other	Urban/ Rural NH	Venue for interview	Quitted working in NH	Interview tools
*FG 1*	*3*				*U/R(mix)*	*NTNU*		*IG*
*FG 2*	*2*	*2*			*R*	*NH*		*IG*
*FG 3*	*2*	*2*			*R*	*NH*		*IG*
*FG 4*	*1*	*2*	*2*		*R*	*NH*		*IG*
*II 1*		*1*			*U*	*NTNU*		*IG + C*
*II 2*		*1*			*U*	*NH*		*IG + C*
*II 3*		*1*			*U*	*NH*		*IG + C*
*II 4*			*1*		*U*	*NTNU*	*X*	*IG + C*
*II 5*	*1*				*U*	*NTNU*	*X*	*IG + C*
*II 6*		*1*			*U*	*NTNU*	*X*	*IG + C*
*FG 5*	*3*			*1(SE)*	*U (mix)*	*NTNU*		*IG + C*
*II 7*				*1(SW)*	*U*	*NTNU*	*X*	*IG + SI*
*II 8*	*1*				*U*	*Home*		*IG + SI*
*II 9*		*1*			*U*	*Online*		*IG + SI*
*II 10*		*1*			*U*	*Phone*		*IG + SI*

*FG; focus group, II; individual interview, RN; registered nurse, LPN; licensed practical nurse, Ass; assistant, SW; social worker, SE; social educator, U; urban, R; rural, NH; nursing home, NTNU; Norwegian University of Science and Technology*, *mix; mix of informants from different nursing homes, IG; semi-structured interview guide, C; case-descriptions, SI; survey instrument*

### Data analysis

Initial analyses were undertaken by the first author by line -by-line coding using pen and paper. The first four focus group discussions were also coded by the second and last author to ensure credibility. Coding was guided by the CGT framework; hence using initial, focused and theoretical coding. We used constant comparisons to find consistencies and differences in the data, thus continually refining concepts and theoretically relevant categories. An audit trail was ensured through writing field reports and memos during the data-collection and analysing phase. NVivo software version 20 was used to assist with the data organisation and coding process. An example of the coding process is presented in Table 2.

**Table 2:** An example from the coding process

Initial codes	Focused codes	Sub-category	Category
Experiencing conflicting needs Ranking care-duties Neglecting least important tasks/care Needing to rationing care Prioritizing physical over psychosocial needs Using time on documenting over patient-related activities Needing to do practical tasks/non-care duties Having to ignore resident asking for help	Prioritizing important/life-saving care Being task-oriented Needing to accomplish non-care duties Ranking residents needs Rationing basic care Down-prioritizing individualized care	Ranking and rationing of care	Normalizing missed care
Experiencing overwhelming workload Lacking time to meet residents wishes/needs Lacking staff Management focusing on cost-cutting Management ignoring concerns from staff Management ignores lack of resources Experiencing little support from management Blaming management for neglected care	Describing demanding work-conditions Feeling continuous lack of time Lacking resources Feeling neglected care is out of their control Lacking support from management	Nursing under unavoidable and unfortunate circumstances	
Lacking knowledge of routines Lacking knowledge about residents Lacking competencies about procedures Experiencing inadequate observations Describing delayed treatment Working with unexperienced staff Working with new and unknown staff Having to assist unskilled staff Needing to follow-up agency-nurses	Staff lacking knowledge and competency Working with unexperienced or unfamiliar staff Experiencing inadequate skill-mix		

Our initial analysis indicated that the same themes reappeared frequently, and it took more time than expected to get to the core of the questions. Acknowledging the sensitivity of the research topic, we continued with individual interviews to see if this allowed participants to go deeper into the relevant themes, and potentially share more sensitive information.

The first three individual interviews gave similar results as the focus groups discussions. Hence, in accordance with the CGT approach we selected the next participants to get more variation in our sample. We eventually also introduced case-descriptions and examples of neglect from a survey instrument on elder abuse as a more casuistic approach to what had previously been a more conceptually guided questioning. This proved fruitful, enabling us to generate more conceptual categories.

We continued with a more focused sampling, directed towards more specific participants including individuals no longer working in nursing homes or participants having some information of the topic and who were reached without institutional gatekeepers. Thus, these participants enabled the deepening of the concepts developed from our data.

After five focus groups discussions and ten individual interviews we came to a point when gathering more data did not seem to create new properties, or further insight about our categories *Neglecting neglect* and *Normalizing missed care*. Further steps towards theoretical sampling and development of substantial theory were hampered by the lockdown situation. We subsequently decided that our data and our analysis were sufficiently developed as abstract analytic categories, although coming short of the criteria for a more formalised theory.

## Results

Examples of neglect were described in all our focus groups discussions and individual interviews, but not necessarily in a very explicit manner. To an extent this reflects that the English research terminology in this field does not necessarily resonate well with the terminology used by practitioners in Norwegian nursing homes.

### Legitimizing neglect in nursing homes

We will henceforth present some ways nursing staff make not fulfilling basic care needs a liveable and doable part of their everyday practice in nursing homes. In order to make neglect an acceptable practice, nursing staff applied different strategies. These strategies were identified as when nursing staff legitimize it by [1] *neglecting neglect*, when the staff are not recognizing their own behaviour as neglectful, as expressed in their actions and language and [2] *normalizing missed care* when resources are lacking and nursing staff are rationing care.

### Neglecting neglect

The category Neglecting neglect has two sub-categories; Undisputable vs. negotiable neglect and Trivializing neglect.

#### Undisputable vs. negotiable neglect

Our first major finding is that there exists a set of explanations making neglect possible in nursing home practice, demonstrating that neglect is actively constructed by the nursing staff as part of a social practice. What qualifies as neglect depends largely on their interpretation of the situation, rather than as a dichotomous division of practices as either neglectful or acceptable. Whereas some cases of neglect are indisputable due to the seriousness of outcome, other cases are open to cultural and individual interpretation, and are thus negotiable. As a consequence, there are forms of neglect that represent legitimate forms of nursing practice, either because of uncontrollable circumstances or instances where what is identified as neglect depends on a number of qualifying conditions. The same action may be presented as adequate in one situation and inadequate in another. Some wishes more discussions and reflections among colleagues about what care they provide.

*“We should perhaps discuss it more. What is good care, what is not good care, and what does it take to be a deviation? If we have a common understanding of this it would be easier to stay within (good care) and to know what should be reported as deviation.”(FG; RN 7)*.

A care culture with strong focus on efficiency and following routines was depicted, leaving little, if any time to give individualized care, having to focus on the basic needs. However, lack of time also made them “cut corners” in covering basic needs to maintain efficiency. When there is lack of time, patient care becomes just another task to be done, to tick off the list, and the nursing home becomes a “care factory”.

*“I experience that the nursing home has become a care-factory, I keep thinking of this. It’s kind of like “swosh-swosh”, then you’ll be done with this and done with that- and finally when you’ve been working for many years I think you become a little emotionally blunt.”(II; RN 13)*.

Neglected care activities were usually put on the to do-list, and postponed until later, although staff members were well aware that postponing the activity most likely meant that it would not be carried out at all.

*“If you postpone it, most of the times when such things are postponed, it ends up not being done.” (II; RN 9)*.

As a consequence, the accumulated effect of neglected activities, might represent a greater risk of unwanted consequences than each single episode alone.

Explicitly intentional neglect as described as a part of elder abuse, in terms of direct harm, denying residents sufficient amounts of food and drink, giving too much or too little medication, or threats of withholding care, was uncommon in nursing homes but recognized as something they “just read about in the papers.” Direct experiences came in the form of people signing for procedures that were obviously not performed and finding colleagues asleep in a resident’s room. An increased awareness of this phenomenon, fuelling questions about the real frequency of the problem, even in their own working place.

#### Trivializing neglect

A trivializing effect was also achieved by describing neglect through the language of missed care, as forgotten or missed basic care needs. This was achieved through a downplaying of the seriousness of their consequences.

*“Did something happen in the evening, is that why it is forgotten? Because it’s easy to forget, after all we are just human.” (II; LPN 7)*.

The word «neglect» implicated seriousness and was given a strong negative value, whereas missed care activities were less so, characterised as something that had been forgotten, that the staff did not have time to do or were too stressed to do.

*“ … neglect sounds more conscious. Then you know what you are doing. It almost sounds like it’s intentional. While deviations and everything else are a little out of your control, that it may not really be as much your fault.” (II; RN 9)*.

The terms like forgotten or missed care were easier to use than neglect or mistreatment which were described as much more negatively loaded.

*“… it gives me a feeling that I did something I should have done better- every single day.” (II; LPN 10)*.

Participants ranked neglect more serious than missed care, but during the interviews some participants experienced a revelation when they recognized that consequences of neglect and forgotten or missed care are much the same.

### Normalizing missed care

The category Normalizing missed care has two sub-categories; Ranking and rationing of care and Nursing under unavoidable and unfortunate circumstances.

#### Ranking and rationing of care

Pointing to situations characterised by limited resources was a common initial response during focus group discussions and individual interviews. These conditions presented nurses with a situation where adequate care was not always an option, forcing them to make the most of the resources available.

*“Two night-shifts ago, I had to provide medicine for residents on three wards. I do not focus on [putting on] hearing aids or glasses- you understand?” (II; LPN 7)*.

Although external conditions influenced nursing in important ways, neglect was still the outcome of deliberate choices made by the nursing staff. These choices represented a form of implicit rationing of care, as limited resources resulted in a state of constantly occurring conflicts, related both to many imposed extra tasks outside care and urgent tasks of equal importance. The guiding principle behind this rationing was the perceived seriousness and immediacy of the consequences of neglecting certain needs.

On top of the hierarchy were life-preserving tasks, that only met competition from equally important tasks, like ordering medicines on time to secure the provision of vital treatment. It was generally accepted that prioritizing basic physical needs such as feeding, washing, toileting, medicine administration and procedural task are ranked as more important than psychosocial needs such as socializing and comforting the resident, as time for chatting and meeting the residents’ psychosocial needs were given the lowest priority.

*“If they [managers] ask “Why did you not make that [phone]call” and I replied “I chose to sit down and talk to her a while instead.” Then it is not quite [laughs] accepted then. In a way, there are more consequences if other things are not done… That’s my feeling.” (FG; RN 1)*.

Tasks related to psychosocial needs also fell below practical tasks that were prioritised to enable the staff in performing care related tasks. Like doing the dishes after breakfast to be able to serve lunch or washing the residents’ clothes and other equipment to be able to feed, dress, and care for the resident. Ranking of tasks thus frequently lead to favouring practical tasks over individualized care.

*“I have learned that we [the nurse] should focus on the residents wishes and support their functions [when doing morning care], but other are more stressed and just- “no, we have to finish now because breakfast is served.” (FG; A 2)*.

Although the lack of time and resources was the dominant narrative in our data, there were also participants questioning this. It was suggested that it is created by the nursing staff themselves, through unrealistic expectations when it comes to standards of care and work capacity.

#### Nursing under unavoidable and unfortunate circumstances

Limited resources provided a plausible reason for neglected care, making it commonly accepted that some care tasks are forgotten, ignored, and missed. The nursing staff’s capacity to cover the residents’ basic needs could thus not be taken for granted. Even if the staff knew what to do, they demonstrated a generous acceptance of acts that also could have been classified as missed care.

*“It is allowed to say: “I have not been able to follow up.” No one is criticized for that. Because we [the staff] know that you do your best with the resources you have. So, it is quite accepted that you forget or don’t follow up tasks.” (FG; RN 1)*.

Situations where work demands were greater than available resources lead to frustration and despair, as ideals and reality collided when the staff was unable to provide the care they wanted. Unable to meet the everyday demands in the nursing home, they adapted a pragmatic attitude, accepting that ideals cannot be met. Without self-blame, they resigned and accepted that many care duties were omitted and missed.

*“When we need more staff or someone who can help, and this is not taken into accounts [by the manager]. I believe that the majority of those who work there see this as neglect by the management- it is not the staff who neglect work tasks- it is the management who does not see this as important enough.” (II; RN 9)*.

These omissions represented a survival strategy in answer to limiting and unavoidable circumstances, leaving them no choice if they were to continue working at the institution.

Although acknowledged as a major source of forgotten and missed care, the influence of the resource situation also played out when it was not necessarily unavoidable, but unfortunate. Unfortunate because of the wrong mix of qualities in available resources. This happened in situations of staff shortages, but also when there were enough hands (sufficient staffing) but not the right heads (adequate skill-mix).

*“Did a resident have to wait for help longer than necessary? That is, it is longer than necessary, but not because we want it, but it is because we are not able to do more than what we do [at the moment].” (II; RN 13)*.

Shortage of staff with formal education, lead people to perform tasks they were not formally trained for. Inexperienced colleagues and colleagues unfamiliar with the workplace also represented challenges. This could happen when working with agency nurses having sufficient formal competence, but limited knowledge about the nursing home and the residents they cared for, resulting in omissions or delays of important observations or treatments. Hence, neglect could occur when highly skilled carers unfamiliar with residents’ baseline states did not discover deterioration in the residents´ physical condition.

*“She [a dement resident with diabetes] was developing hypoglycaemia, but was not able to tell [the carer]. Thus, it is so important to know them [the residents] well. What is being overlooked because they do not know them?” (II; LPN 7)*.

Care was also forgotten or missed as new or inexperienced staff were not familiar with residents, routines etc., thus not being able to individualise care in a way the regular staff would do. Hence, a normal state existed, wherein the experienced staff push their limits to cover up for more inexperienced and unskilled staff.

*“The need for more competent staff is not made visible because the regular employees go a long way [to cover it up] and are dutiful.” (FG; A 1)*.

Concerns regarding inadequate staffing and skill-mix were not always heard by management. These unfortunate circumstances bolster a nursing practice which serving to make neglect invisible.

## Discussion

This study is based on the fact that neglect, as defined by researchers, is common in Norwegian nursing homes [4]. Prior research presents a numerous examples of and causal explanations for neglect happening in nursing homes based on the outsiders’ perspectives on this phenomenon [14, 35–41]. We add to this knowledge by studying the meaning making efforts of the nursing home staff. A crucial and initial finding was that the concept of neglect was not a part of the everyday language of the nursing home, although the phenomena identified by the same concept in the academic literature were described by the participants. Furthermore, our analysis explains neglect in nursing home as an outcome of nursing staffs´ construction of a social world in which neglect becomes a liveable practice, by *legitimizing neglect* and their own neglective practices. Neglect is thus made doable and allowed to continue in nursing home through different strategies, such as neglecting neglect and normalizing missed care. Hence, neglect becomes liveable and doable in nursing home settings when nursing staff makes neglect an option in their nursing practice and legitimize neglect through *neglecting neglect* and *normalizing missed care*.

This legitimizing of neglect is an option in part related to a material world presenting unavoidable and unfortunate circumstances the nursing care are delivered under, where there is a strong focus on efficiency and need for rationing of care. However, part of this is also related to the social construction of nursing as a caring profession, and their response to not being able to fulfil nursing home residents´ basic needs. Accepting neglect or making neglect an option/excusable is a way of making their care failures liveable for themselves, thus makes them able to continue to work at the nursing home under the existing circumstances.

This construction of the nursing home reality may thus in no little way be encouraged by a lack of viable options, which at the extremes may include condemnation of their own practices, quitting their jobs and/or taking political activism. Rather than facing such severe consequences, they are adapting through making neglect doable when it has no immediate and severe consequences for the patients, no immediate negative consequences for staff members, is attributed to factors outside the staff’s control, or the consequences for patients are constructed as minor and unavoidable. These findings do not comprise an objective reality, but is the reality constructed by nursing home staff, believing that these are issues that need to be better understood if improvements are to be achieved. They are real in their consequences, posing problems for both patients and staff members. We start off our discussion by looking at what the problem is presented to be.

### What kind of problem is neglect presented to be?

To study what the problem is presented to be is interesting [48]. Our analysis indicates that neglect is a topic that is not easily explored in the nursing home setting in an explicit and spontaneous manner, perhaps reflecting a social taboo which is not openly talked about. Without eliminating this possibility totally, the participants’ responses prompted by case histories and survey examples depict a topic that can be explored through a casuistic approach rather than a conceptual one. Defined as a social practice, it may also be beneficial to study neglect through an ethnographic approach.

As described in the introduction and replicated in our data, neglect is not simply a thing out there, but part of a reality that is open to interpretation and negotiation, framed by discourses. The health policy discourse in past decades has emphasized the responsibility of the individual health worker. There has been a tendency to blame the person (resident or employee) rather than structural factors. A culture where one is looking for someone to blame, is also a culture where employees can be reluctant to reveal information about threats to the quality of care, to protect colleagues and themselves from punishment [49]. This may have been particularly true when neglect has been paired with words like abuse and perpetrator, as part of a process allocating blame and questioning individuals’ moral qualities.

Although there are examples of neglect bordering on negligence and criminal offence, our analysis demonstrates that behaviour that come within formal definitions of neglect are not the hallmark of a few questionable staff members, but an integrated part of ordinary practice in nursing homes. Rather, it may be understood as a social practice upheld by a lack of recognition as a problem, and as a problem presented as an unavoidable or escapable one when recognized.

What for some can be seen as condemnable behaviour, is seen as excusable behaviour by others. In the context of nursing home care, structural factors in the form of lacking or inadequate resources, serve to free individuals of responsibility, thus making neglect excusable. Accordingly, there are studies demonstrating that nurses do not consider themselves individually responsible for neglectful actions when the problem can be defined as primarily structural/institutional or a leadership/political problem [36, 50–52]. Other questionable behaviours have also been made excusable when attributed to challenging resident behaviours. Such causal attributions are thus important to determine if a behaviour is seen as neglect or not [12].

### Rationing of care – from implicit and individual to explicit and institutional?

We found that nursing staff make active rationing of care in their everyday practice, basically demonstrating that when resources are scarce, services provided are restricted to the lower levels of Maslow´s hierarchy of needs. These findings may thus be seen as another example of the situation described by Scott and colleagues, where decisions about rationing are covert and left to individual nursing providers [23]. Our findings thus resonate a reality found across nations, thus supporting their generalizability.

Rationing of care takes place in many health care settings in Norway [5, 53, 54], commonly presented as an unavoidable part of health care provision even under the best of circumstances. It is an issue that has been addressed nationally in recent decades, resulting in national guidelines based on explicit criteria for rationing of care that are ethically guided [55]. Unfortunately, this work has not benefitted the care of the elderly, indicating that there is still work to do.

The resulting consequences may not only have direct adverse effects for patients, but also indirectly through its effect on members of the nursing staff. This is argued by Ball, who address what happens to nurses when they are unable to deliver necessary nursing care, having to make compromises on the quality of care given, struggling to maintain professional and personal values and standards. When the ideal is crushed, it may affect nurses’ job-satisfaction, subsequently influencing the quality of care given [25]. Hence, there is a need to create conditions under which nursing staff can thrive and develop, in order to be able to deliver high quality care, rather than environments where the lack of resources cause burn-out, leaving the profession or adaption to insufficient care levels [5, 25, 56, 57].

### Legitimizing neglect in nursing homes

We found neglecting neglect and normalizing missed care as two ways of legitimizing neglectful practice. They provided opportunities for manoeuvring the moral landscape that is normally framing nursing practice, by enabling nursing home staff to define what would otherwise be seen as neglectful practices as excusable ones. This way of constructing reality has also been found in studies that have identified an increased *tolerance* for elder neglect, thus making it culturally accepted [12, 58, 59]. These tendencies might be interpreted as the nursing staff´s attempt to maintain the depiction of themselves as caring.

What could otherwise be seen as neglectful behaviour is thereby constructed as omissions, as when forgetting something is seen as an example of an unintentional general flaw in human nature. Like lack of resources, this flaw is attributed to be outside the nursing staff’s control. In terms of representing a “missed care discourse” this represents a way for the carers to handle not giving necessary care without challenging their personal qualities or attitudes [25]. This discourse thus allows for normalizing missed care as a part of the existing working culture. It has thus been argued that operating within this discourse enables them to continue going to work without feeling guilty, get burned out or quit, which may not have been as easy if the “neglect discourse” framed the situation. The consequences for the residents, however, remain the same, regardless of the language used to construct a “truth” about care who is inadequate or neglected or missed. If the result is the same, it might not matter if we call it *neglect* or *missed care.*

Our findings illustrate that both the *neglect* and *missed care* label can be used interchangeably for the same acts of omission. This is also reflected in the research literature, where some researchers label an episode where the resident did not receive oral care in many days as neglect, others call it missed care.

A more radical interpretation of the research findings is that the nursing profession is blind to the existence of routine neglect through delivery of “shitty nursing”. It is suggested that poor and inadequate nursing care may not be the exception, but the new normal [59]. Neglect may thus be seen as a product of a nursing home culture where neglect is legitimized by neglecting neglect and normalizing missed care. Presenting the problem as a cultural one has already been done in the wider context of patient safety literature [26, 27]. By doing so, it has been acknowledged that the problem is a complex and multifactorial one, replacing earlier representations of the problem as one basically related to individual moral. This may prove a more fruitful way forward, but albeit not necessarily an easy one.

### Strengths and limitations

This is not a fully fledged grounded theory study, in the sense that no substantial theory has been developed [15]. Sampling for this study has been challenging, as recruitment for the focus groups have depended on institutional arrangements, meaning that accomplishing a purposeful mix of participants have been outside our control. We have still accomplished to recruit a diverse sample with regard to education, background, working experience, and negative cases by way of recruiting individuals that have chosen to leave nursing home practice. The Covid lockdown has not helped our recruitment, and we have thus been unable to perform the theoretical sampling that is required to reach the ultimate goal of a CGT study. In addition, we acknowledge that differences in patients` characteristics, staff factors and environmental factors (including staff culture) in the 17 nursing homes we recruited from represent a source of variation that we all have not been able to study in a systematic fashion.

Having said this, the study has benefitted from the application of an iterative process rather than a purely inductive one. It is to our knowledge the first study exploring neglect as constructed by nursing home staff in Norway. Despite its theoretical shortcomings, it thus adds knowledge from a perspective hitherto not thoroughly investigated. The study may thus also serve as a steppingstone for more theoretically guided explorations in its field. It has furthermore benefitted from the cooperation of a research team consisting of individuals with diverse disciplinary backgrounds.

## Conclusion

Neglecting residents’ needs in nursing homes is a preventable and unnecessary burden in elder care. Our findings show that the nursing home staff need to construct a liveable and doable work reality for themselves by legitimizing neglect, through neglecting neglect and normalizing missed care. This study shows how acts of neglect can continue to occur and gives important insight in the processes that takes place in nursing home practice. More research about what impact language and social culture have in constructing realities in this subject of matter, might lead to knowledge necessary for changing the perception of quality of care in nursing homes. If this is not addressed, nursing care might continue to be compromised, leading to suffering for both caregivers and residents in nursing homes in the future.

## Electronic supplementary material

Below is the link to the electronic supplementary material.

Supplementary Material 1

## Data Availability

The dataset generated from the study will be publicly available upon reasonable request to the corresponding author once anonymization of the data has been completed.

## Declarations

### Authors’ information (optional)

SBL is doing this research as part of her dissertation research. WKM and LM have long experience in the research field of elder abuse and neglect, whereas JAS have contributed with his experience in qualitative research.

## Abbreviations

CDC: Centers for Disease Control and Prevention
CGT: Constructivist grounded theory
FG: Focus group
II: Individual interview
LPN: Licensed practical nurse
NH: Nursing home
NSD: The Norwegian Centre for Research
RN: Registered nurse
SE: Social educators
SW: Social workers
