# Supplementary material for: Legitimizing neglect - a qualitative study among nursing home staff in Norway
Source: BMC Health Serv Res. 2023 Mar 6;23:212. doi: 10.1186/s12913-023-09185-1 (PMC9990246; doi:10.1186/s12913-023-09185-1)
Supplement: Supplementary file 1 — Supplementary Material 1 [file 12913_2023_9185_MOESM1_ESM.docx]

# Interview-guide

## Main- question

How do you perceive and reflect on neglect?

Introduce case studies or questions from survey-instrument if delayed response, or difficulties to understand the topic.

## Follow-up questions

• What is the first thing that comes to your mind when inadequate follow-up of nursing home resident's care needs is mentioned?

• What types of action/lack of actions can this be?

• What experiences do you have with neglect?

• What are signs/consequences of neglect?

• What different types and possible levels of neglect do you think exist?

• What do you think is/can be reasons for neglecting residents in nursing homes?
